# Towards the early detection of ductal carcinoma (a common type of breast cancer) using biomarkers linked to the PPAR(γ) signaling pathway

**DOI:** 10.6026/97320630015799

**Published:** 2019-12-09

**Authors:** Ghazala Sultan, Swaleha Zubair, Iftikhar Aslam Tayubi, Hans-Uwe Dahms, Inamul Hasan Madar

**Affiliations:** 1 Department of Computer Science, Aligarh Muslim University, Aligarh, Uttar Pradesh 202001, India; 2 Faculty of Computing and Information Technology, Rabigh, King Abdulaziz University, Jeddah 21911, Saudi Arabia; 3 Department of Biomedical Science and Environmental Biology, KMU-Kaohsiung Medical University, Kaohsiung, Taiwan; 4 Department of Biotechnology, School of Biotechnology and Genetic Engineering, Bharathidasan University, Tiruchirappalli, 620024, Tamil Nadu, India

**Keywords:** Breast Cancer, ductal Carcinoma In-situ (DCIS), invasive ductal carcinoma (IDC), PPAR(γ)signaling pathway, microarray, biomarker Discovery

## Abstract

Breast cancer is a leading cause of morbidity and mortality among women comprising about 12% females worldwide. The underlying alteration in the gene expression, molecular mechanism and metabolic pathways responsible for incidence and progression of breast tumorigenesis are yet not completely understood. In the present study, potential biomarker genes involved in the early progression for early diagnosis of breast cancer has been detailed. Regulation and Gene profiling of Ductal Carcinoma In-situ (DCIS), Invasive Ductal Carcinoma (IDC) and healthy samples have been analyzed to follow their expression pattern employing normalization, statistical calculation, DEGs annotation and Protein-Protein Interaction (PPI) network. We have performed a comparative study on differentially expressed genes among Healthy vs DCIS, Healthy vsIDC and DCIS vs IDC. We found MCM102 and SLC12A8as consistently over-expressed and LEP, SORBS1, SFRP1, PLIN1, FABP4, RBP4, CD300LG, ID4, CRYAB, ECRG4, G0S2, FMO2, ADAMTS5, CAV1, CAV2, ABCA8, MAMDC2, IGFBP6, CLDN11, TGFBR3as under-expressed genes in all the 3 conditions categorized for pre-invasive and invasive ductal breast carcinoma. These genes were further studied for the active pathways where PPAR(γ) signaling pathway was found to be significantly involved. The gene expression profile database can be a potential tool in the early diagnosis of breast cancer.

## Background

Breast cancer is one of the second leading cause of mortality in females at the global level [1]. Around 2 million incidences of breast cancer had been reported in the year 2018 [2]. Earlier studies have reported that the frequency of registered cases has increased alarmingly in the rural areas as compared to incidences in the urban area [3-5]. The estimated incidence rate of various types of cancer by the year 2030 has been predicted to be 1.7 million with the expectation of 17 million deaths per year [6,7]. Not all cancers are considered to be fatal; rather it is treatable if diagnosed at an early stage. Breast cancer has been considered as a complex cancer type due to its heterogeneity [8], having a wide range of risk factors involved, including a high-fat diet, alcohol intake, obesity, genetic risk and family history, etc. [9].

Breast cancer can be broadly classified into two categories based on the location of tumor origin in the breast, specifically ductal and lobular carcinomas [10]. A ductal tumor develops in ducts, which contributes to approximately 80% of reported breast cancer cases. The second most commonly found category is a lobular tumor, which develops inside the breast lobules and found in 10-15% of diagnosed tumors [11]. Among the two major categories of breast tumorigenesis, ductal tumor has been more widely studied and provides deeper insight into molecular mechanisms and genetic basis of the disease. Ductal Carcinoma In-Situ (DCIS) is pre-invasive type breast cancer with an alarming increase in frequency from 1-5% to 10-15% in recent years [12]. It can be treated if detected earlier, before abnormal proliferation of cells inside the ducts, but if not diagnosed at an early stage, it may progress abruptly to other parts termed as Invasive Ductal Carcinoma (IDC), the advanced stage of DCIS. Various clinical methods for breast examination include mammography, MRI, ultrasound and breast screening. With the increment in breast cancer screening programs, the number of DCIS cases to be diagnosed is expected to increase exponentially [13]. In general, breast cancer can be diagnosed by physical examinations and changes that start appearing from the very early onset of cancer progression. Top five most common symptoms of primary breast cancer include breast lump (81.4-84.5%), nipple abnormalities (5.9-7.9%), breast pain (5.5-7.5%), breast skin abnormalities (1.5-2.6%) and axillary lump (0.7-1.6%) [14].

Breast cancer heterogeneity among patients can be classified into luminal and basal-like immuno histo chemical profiles based on estrogen receptors (ER), progesterone receptors (PR), and human epidermal growth factor receptor (HER2). On comparison based on characteristics and clinical behavior, HER2 positive (HER2+) DCIS are considered more violent than any other DCIS subtypes and studied as a potential biomarker for progression to invasive breast carcinomas whereas at cellular level CD44/CD24, N-cadherin/E-cadherin and CD74/CD59 were reported as the biomarker pairs for specifically in DCIS cases [15-17].

**Figure 1:**
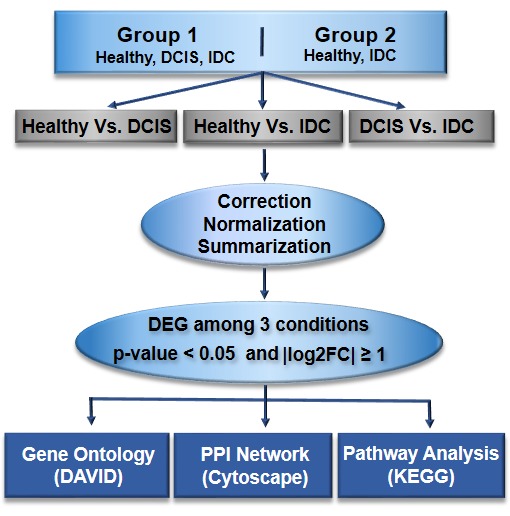
Overall schematic diagram towards the early detection of ductal carcinoma

Statistically, in all the diagnosed cases, 90% of BC is due to an aberrant mutation in various genes and 5-10% is due to the dysfunctional genes inherited from parents [3]. Around 2,948 genes have been reported to be involved directly or indirectly in breast cancer development, in which 466 plays a crucial role in the development of cancer [18]. The genes, which are potentially responsible for breast cancer, are BRCA1, BRCA2, PTEN, ATM, BARD1, BRIP1, CHEK2, PALB2, RAD50, etc. In general, gene expression profiling has a significant role in the early detection of breast cancer. Gene expression analysis can help us get better predictive as well as prognostic strategies [19,20]. Thus, it is of high relevance to perform gene expression analysis to identify DEGs in cancerous cells as compared to healthy cells. Microarray-based gene expression analysis has opened a new horizon in cancer biology and has exceptionally provided aid to our knowledge about the complexity of BC tumorigenesis [21]. Microarray analysis helps in genetic alteration that might be responsible for cancerous alterations by calculating genes expression values for the respective disease under study.The study schema is given in (Figure 1)

## Methodology

### Affymetrix microarray data:

The breast cancer-specific microarray data sets were downloaded from GEO (Gene Expression Omnibus https://www.ncbi.nlm.nih.gov/geo/) database with the accession id GSE21422 and GSE5764. The microarray data in cell file format is based on the GPL570 [HG-U133_Plus_2] Affymetrix Human Genome U133 Plus 2.0 Array platform for both the datasets. The downloaded dataset with accession ID GSE21422 (set-1) consists of 3 sample conditions from a healthy subject, DCIS and IDC and the dataset with accession ID GSE5764 (set-2) consists of Normal ductal cells and IDC samples. DCIS being the primary or early stage of breast cancer for tumorigenesis, in current analysis we selected three stages, i.e. healthy, DCIS and IDC patient gene expression with the aim to identify a set of genes which play a pivotal role in the initiation, progression of cancerous and also gene involved in further development of the DCIS to IDC stages.

### Data pre-processing:

The microarray data analysis was carried out to identify potential marker genes related to breast cancer development in different stages as stated above. It enabled us to identify a set of genes that play an essential role, which acts as a tool for early detection of Ductal Carcinoma. From set-1 and set-2, we included nine samples for each condition and CEL files were downloaded for further processing. Thus, the three different cases under this study are gene expression analysis of DCIS vs healthy samples, IDC samples vs healthy and DCIS with IDC. The sample array was analyzed in R (3.2.5) using microarray data analysis specific Bioconductor packages, which are freely available in Bioconductor (https://www.bioconductor.org/), like limma, affy, and few others in-house built pipelines for microarray data analysis. Normalization of data was done using Robust Multi Array Analysis (RMA) procedure, which uses quantile normalization to minimize the noise impact. RMA provides precise estimates of probes expression as it performs various steps, which include background correction, normalization of probes and summarization. Normalization of expression data brings data closer and makes them less scattered, which helps to balance data to make meaningful biological comparisons [22]. Gene expression for 19,902 genes obtained as the result of the pre-processing of both the datasets. The set of probe IDs with no corresponding locus or gene IDs were excluded for further processing.

### Differentially expressed genes (DEGs) analysis: 

The differentially expressed genes from the DCIS patient compared with those from the healthy people were analyzed by limma package (v.3.26.8) and then the t-test was applied to the data to get the final list of significantly expressed genes table. The extraction of a gene on DEG based on the criteria limited to adjusted p-value < 0.05 and |log2FC| as 1, which was performed based on t-test on the resultant normalized data. P-values were adjusted by applying Benjamini and Hochberg (FDR).

### Gene ontology (GO), annotation and pathway enrichment analyses:

The Database for Annotation, Visualization and Integrated Discovery (DAVID), an open-source tool for Gene Ontology (GO) annotation of differentially expressed genes is used for gene-gene annotation. GO terms are categorized into biological processes, cellular components and molecular function where biological process refers to complex changes on the granularity level of the cell which is mediated by one or more gene products, cellular component is a part of cell or its extracellular environment which may contain gene product and Molecular Function help us to understand the potential of the molecule to execute the function [23], which may be actively responsible in gene expression variation. Pathways in which the DEGs are involved were identified using KEGG (Kyoto Encyclopedia of Genes and Genomes).

## Discussion

**Figure 2:**
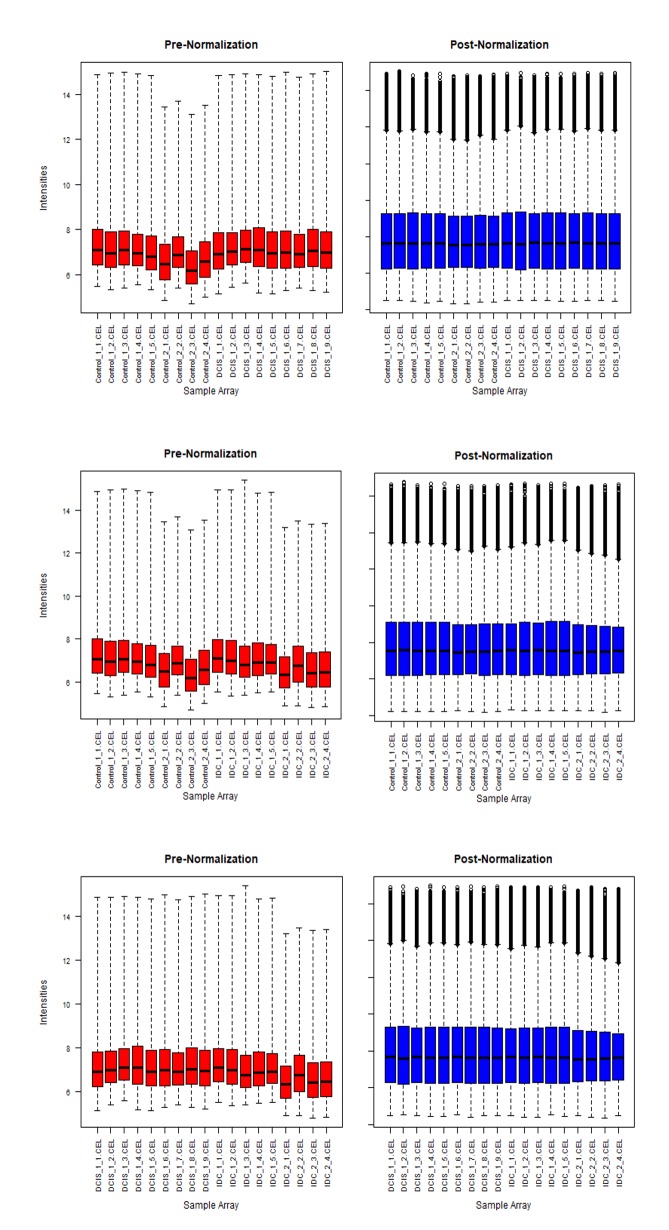
Pre-normalization (raw intensity of genes) and post-normalization (intensity after normalization) state for 3 different comparison categories (a) Healthy vs DCIS, (b) Healthy vs IDC and (c) DCIS vs IDC samples.

The pre- and post- normalization hybridization intensities across all the samples in the dataset has been shown in (Figure 2). The pre-normalization plots show non-uniform data distribution. The intensity of the data was adjusted using quantile normalization algorithms. The expression values were determined using Affy package in R software. Thus the distribution of data post normalization has uniform intensity within the same intervals and the same density center. This helps us to understand the non-uniformity of the gene expression in the raw data, which made it necessary to normalize the data before processing for minimizing the error probability in the results.

**Figure 3:**
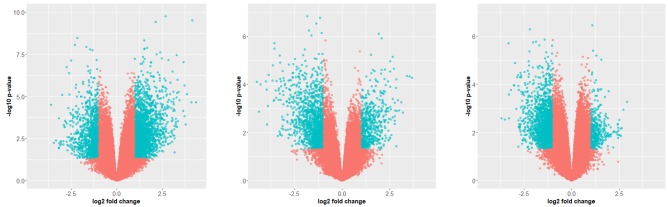
The significantly expressed genes (p value <0.05) having logFC >1 representing upregulated genes and logFC < -1 corresponds to downregulated genes for (a) Healthy vs DCIS, (b) Healthy vs IDC and (c) DCIS vs IDC.

The expressed genes with the threshold value for log2 Fold Change (logFC~1) used to obtain the DEGs in various conditions that are Healthy vs DCIS, Healthy vs IDC and DCIS vs IDC. Each circular dot represents one gene with its corresponding -log10(P-value) and the cut-off for the selected DEG represented in blue circular dot highlighting the most significant genes which fall under the category of differentially expressed genes. LogFC > 1 representing upregulated genes and logFC < -1 corresponds to down regulated genes (Figure 3). This method leads to filter the genes on the scale of unstandardized signals (e.g. log2fold change) against noise-adjusted/standardized signals (e.g., log10(p-value)) and help us determine the curated set of genes which are significantly expressed. It also helps to visualize those genes to present them more interactively.

## Pathways analysis:

The PPARs family is a fat metabolism related pathway which includes PPARα, PPARγ and PPARδ, serving different functions in cancer categorized as PPARα and PPARγ inhibits tumor progression while PPARδ promotes tumor development [24]. Although the results have not always been consistent but PPARγ, which is hormone receptor and plays role in regulating adipocyte differentiation, insulin-signaling showed associativity with breast cancer risk [25]. Thus, PPAR signaling pathway is considered as actively involved in tumerogenesis. Among the 20 down regulated genes, FABP4/aP2, SORBS1/CAP and PLIN1/Perilipin were significantly enriched in the peroxisome proliferator-activated receptor (PPAR) pathway (Figure 5) and noticeably these genes were down regulated in both pre-invasive (DCIS) and invasive ductal carcinomas (IDC) in our result. Significance of PLIN1 as a gene that inhibits cancer cell proliferation, migration and invasion in human breast cancer is reported in a study where it was also found as significantly under-expressed in mRNA expression [26].

The upregulated genes are SLC12A8 and MCM10, which showed high expression in both DCIS and IDC. An earlier study reports the high expression levels of SLC12A8 is associated with better prognosis of breast and pancreatic ductal carcinoma, therefore considered as a significant gene that contributes to the personalized treatment of breast cancer [27]. MCM10 (Minichromosome Maintenance 10 Replication Initiation Factor) is a protein-coding gene, which was found upregulated in breast tumor tissues and also in the triple-negative breast cancer. MCM10 might induce breast cancer metastasis via the Wnt/- catenin pathway, which defined it as a potential diagnostic tool as well as a promising target for breast cancer [28].

**Figure 4:**
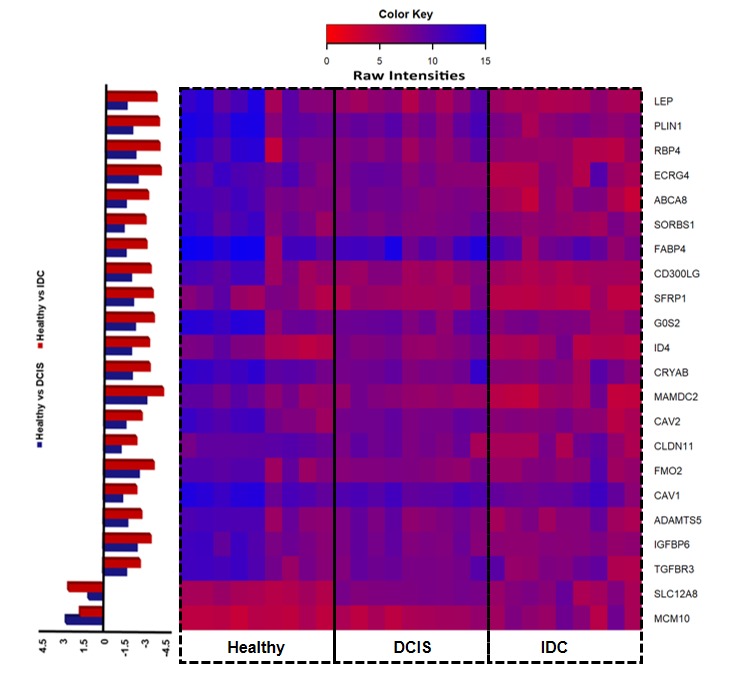
Expression intensity of differentially expressed genes for the entire sample array in the datasets.

**Figure 5:**
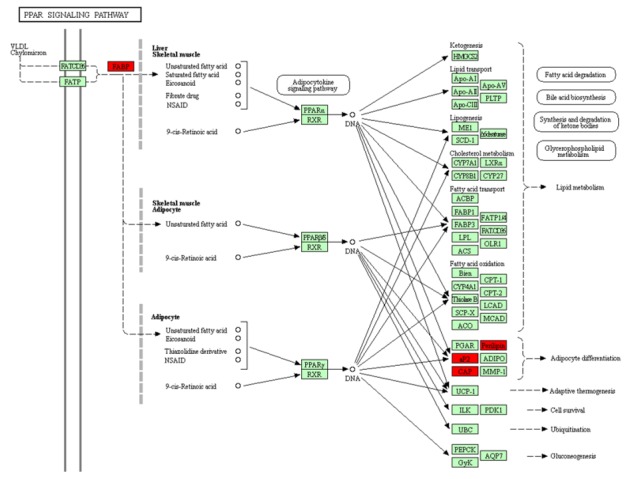
Significantly down regulated genes (from the current study; PLIN1, SORBS1, FABP4, etc) involved in the PPAR signaling pathway is highlighted in red color.

We studied the common genes which are differentially expressed among all 3 conditions i.e. Healthy samples vs DCIS, Healthy vs IDC and DCIS vs IDC. By comparing the first 2 sets we obtained genes that were differentially expressed in both Healthy vs DCIS, Healthy vs IDC. In total, we obtained 308 upregulated and 355 downregulated genes. For this set of commonly expressed DEGs, we studied their expression and compared it with the genes, which were showing consistent upregulation, or down regulation. We obtained 2 upregulated and 20 down regulated genes and filtered them for Gene Enrichment Analysis, Pathway Analysis (Table 1) and Gene/PPI network for 22 differentially expressed identified genes. We found MCM102 and SLC12A8as upregulated genes among groups in all the 3 conditions and LEP, SORBS1, SFRP1, PLIN1, FABP4, RBP4, CD300LG, ID4, CRYAB, ECRG4, G0S2, FMO2, ADAMTS5, CAV1, CAV2, ABCA8, MAMDC2, IGFBP6, CLDN11, TGFBR3as downregulated genes(Figure 4). Some of the identified DEGs were found to be directly related to breast cancer. Also, the identified DEGs such as CAV1, FABP4, FMO2, G0S2, MAOB, PCK1, PLIN1, RBP4 showed close relativity with alcoholism, endometrial and bladder cancer which implies alcohol consumption might be a crucial factor responsible for ductal carcinoma. Additionally, abrupt growth in endometrial cells can lead to malignant tumor growth or endometrial hyperplasia, which can further lead to cancer.

## Conclusion

Gene expression based analysis has been used for disease biomarker discoveryfor a decade, providing ways for better diagnosis, novel drug design strategies and biomarker identification which leads to improvement of clinical treatment efficacy. The expression analysis results reported in the present study can be exploited as a breast cancer-specific novel biomarker and drug target identification. In recent years, increasing incidences of breast cancer demand extensive research on progression mechanisms of this dreadful disease. The breast cancer-associated genes were found to be majorly involved in PPAR signaling pathway, which consists of nuclear hormone receptors playing a different role in tumor development and also in cancer progression. We have identified the genes which are involved in PPARγ pathway, which are which are actively involved inadipocytic differentiation and the evidence of associativity of these genes with breast cancer has also been reported in earlier studies. Thus, PPAR pathway can be considered as having major role in tumerogenesis, which regulates cancer cell proliferation, survival, fatty acid-activated nuclear hormone receptors and its derivatives. Also, we have identified the genes which involved in alcoholism-related pathway and other cancer types which signals these set of genes to be considered as potential genes for early detection of breast cancer and its progression from Ductal Carcinoma In-Situ to Invasive Ductal Carcinoma. Furthermore, this study can be extended to RNA-Seq data from breast cancer patients to validate the existing results and Exome Seq data can also be tested for mutation analysis of the identified significantly expressed genes for better understanding of the underlying biological mechanism in breast cancer occurrence and its progression.

## Figures and Tables

**Table 1:** Genes and pathways related to oncological study for various molecular and biochemical functions.

Pathways	Combined score	No. of genes	Genes
PPAR signaling pathway	399.1485	3	FABP4;SORBS1;PLIN1
Regulation of lipolysis in adipocytes	239.8322	2	FABP4;PLIN1
ABC transporters	69.3766	1	ABCA8
Fluid shear stress and atherosclerosis	68.7799	2	CAV2;CAV1
Focal adhesion	41.1671	2	CAV2;CAV1
Proteoglycans in cancer	40.5699	2	CAV2;CAV1
Adipo cytokine signaling pathway	39.2148	1	LEP
TGF-beta signaling pathway	27.2222	1	ID4
Longevity regulating pathway	22.8477	1	CRYAB
Metabolism	21.0752	8	CAV1,FABP4,FMO2,G0S2,MAOB,PCK1,PLIN1,RBP4
Leukocyte transendothelial migration	20.0145	1	CLDN11
AMPK signaling pathway	18.1365	1	LEP
Insulin signaling pathway	14.9769	1	SORBS1
